# Supplementary material for: Generation and Immunogenicity of Virus-like Particles Based on the Capsid Protein of a Chinese Epidemic Strain of Feline Panleukopenia Virus
Source: Vet Sci. 2025 May 20;12(5):503. doi: 10.3390/vetsci12050503 (PMC12116079; doi:10.3390/vetsci12050503)

Table S1-Screening Ala91Ser FPLV variants in NCBI database

No.	Gene ID	Name of strains	Host	Country	Year	Site 91	Proportion of variant
1	D88287	PLI-IV	Felis catus	Japan	1968	A	0/1
2	AB000056	Obihiro	Felis catus	Japan	1974	A	0/1
3	AB000068	TU4	Felis catus	Japan	1975	A	0/5
4	D78584	TU10	Felis catus	Japan	1975	A	
5	AB000064	TU12	Felis catus	Japan	1975	A	
6	AB000066	TU2	Felis catus	Japan	1975	A	
7	AB000070	TU8	Felis catus	Japan	1975	A	
8	JN867595	TX/Rac2.2/78	Procyon lotor	USA	1978	A	0/3
9	KM624023	TX/Rac3/1978	Procyon lotor	USA	1978	A	
10	JN867596	TXRac1.2/78	Procyon lotor	USA	1978	A	
11	D88286	483	Felis catus	Japan	1990	A	0/2
12	JN867594	NJ/RPV-6/90	Procyon lotor	USA	1990	A	
13	AB000054	Fukagawa	Felis catus	Japan	1993	A	0/1
14	AB000050	94-1	Felis catus	Japan	1994	A	0/3
15	AB000052	AO1	Felis catus	Japan	1994	A	
16	AB000059	Som1	Felis catus	Japan	1994	A	
17	AB000061	Som4	Felis catus	Japan	1995	A	0/1
18	AF015223	T1	leopard cat	China/Taiwan	1996	A	0/1
19	AB054225	V142	Felis catus	Vietname	1997	A	0/3
20	AB054226	V208	Felis catus	Vietname	1997	A	
21	AB054227	V211	Felis catus	Vietname	1997	A	
22	EU498682	198/01	Felis catus	Italy	2001	A	0/1
23	EU498684	103/02	Felis catus	Italy	2002	A	0/2
24	EU498683	41/02	Felis catus	Italy	2002	A	
25	EU498685	150/03	Felis catus	Italy	2003	A	0/3
26	EU498686	189/03	Felis catus	Italy	2003	A	
27	EU498687	30/003	Felis catus	Italy	2003	A	
28	AB262659	SL1	Snow leopard	Japan	2004	A	1/12<br>8.3%
29	EU498688	134/04-1	Felis catus	Italy	2004	A	
30	EU498689	134/04-2	Felis catus	Italy	2004	A	

31	EU498690	134/04-3	Felis catus	Italy	2004	A	
32	EU498691	134/04-5	Felis catus	Italy	2004	A	
33	EU498692	143/04	Felis catus	Italy	2004	A	
34	EU498693	355/04	Felis catus	Italy	2004	A	
35	DQ474237	GT-3	Tiger	China/Guangxi	2004	A	
36	DQ474235	HT-69	Tiger	China/Heilongjiang	2004	A	
37	DQ474236	JF-1	Tiger	China/Jilin	2004	A	
38	DQ099431	JF-3	Tiger	China/Jilin	2004	S	
39	DQ474238	SM-4	Tiger	China/Shaanxi	2004	A	
40	DQ099430	ZF-5	Tiger	China/Henan	2004	A	
41	EU498695	119/05	Felis catus	Italy	2005	A	0/2
42	EU498694	20/05	Felis catus	Italy	2005	A	
43	EU498713	97/06-10	Felis catus	Italy	2006	A	
44	EU498714	97/06-11	Felis catus	Italy	2006	A	
45	EU498696	22/06	Felis catus	Italy	2006	A	
46	EU498715	228/06	Felis catus	Italy	2006	A	
47	EU498707	42/06-12	Felis catus	Italy	2006	A	
48	EU498710	42/06-17	Felis catus	Italy	2006	A	
49	EU498711	42/06-18	Felis catus	Italy	2006	A	
50	EU498697	42/06-G1	Felis catus	Italy	2006	A	
51	EU498705	42/06-G10	Felis catus	Italy	2006	A	
52	EU498706	42/06-G11	Felis catus	Italy	2006	S	2/27
53	EU498708	42/06-G14	Felis catus	Italy	2006	A	(7.4%)
54	EU498709	42/06-G16	Felis catus	Italy	2006	A	
55	EU498712	42/06-G19	Felis catus	Italy	2006	A	
56	EU498698	42/06-G2	Felis catus	Italy	2006	A	
57	EU498699	42/06-G3	Felis catus	Italy	2006	S	
58	EU498700	42/06-G4	Felis catus	Italy	2006	A	
59	EU498701	42/06-G5	Felis catus	Italy	2006	A	
60	EU498702	42/06-G6	Felis catus	Italy	2006	A	
61	EU498703	42/06-G7	Felis catus	Italy	2006	A	
62	EU498704	42/06-G8	Felis catus	Italy	2006	A	

63	KU248456	PT001/06	Felis catus	Portugal	2006	A	
64	KU248457	PT002/06	Felis catus	Portugal	2006	A	
65	KU248458	PT003/06	Felis catus	Portugal	2006	A	
66	KU248459	PT004/06	Felis catus	Portugal	2006	A	
67	KU248460	PT005/06	Felis catus	Portugal	2006	A	
68	KU248461	PT006/06	Felis catus	Portugal	2006	A	
69	KT240128	PT020/06	Felis catus	Portugal	2006	A	
70	EU697383	HT-262	Tiger	China/Heilongjiang	2007	A	4/17 (24%)
71	EU360959	1335/07	Felis catus	Hungary	2007	S	
72	EU145593	389/07	Asian palm civet	Hungary	2007	A	
73	EU498718	443/07	Felis catus	Italy	2007	A	
74	EU498719	490/07	Felis catus	Italy	2007	A	
75	EU498720	498/07	Felis catus	Italy	2007	A	
76	EU498716	50/07-1	Felis catus	Italy	2007	A	
77	EU498717	50/07-2	Felis catus	Italy	2007	A	
78	EU360958	933/07	Felis catus	Hungary	2007	S	
79	EU697387	HT-163	Tiger	China/Heilongjiang	2007	A	
80	EU697384	HT-290	Tiger	China/Heilongjiang	2007	A	
81	EU697386	HT-374	Tiger	China/Heilongjiang	2007	A	
82	EU252145	KF001	Felis catus	South Korea	2007	A	
83	EU252146	KF002	Felis catus	South Korea	2007	S	
84	EU252147	KF003	Felis catus	South Korea	2007	S	
85	KT240129	PT001/07	Felis catus	Portugal	2007	A	
86	KU248462	PT002/07	Felis catus	Portugal	2007	A	
87	GQ857595	BFPV	Blue fox	China/Shandong	2008	A	2/26 7.7%
88	OR566997	RJ9222008	Felis catus	Brazil	2008	S	
89	FJ231389	BJ-22	Monkey	China/Beijing	2008	A	
90	FJ936171	ChangC2007	Felis catus	China/Jilin	2008	A	
91	EU498681	Felocell-Pfizer vaccine	Felis catus	Italy	2008	A	
92	FJ405225	FPV	Tiger	China/Jilin	2008	A	
93	HQ184189	K2	Felis catus	South Korea	2008	A	
94	HQ184193	K22	Felis catus	South Korea	2008	A	

95	HQ184194	K23	Felis catus	South Korea	2008	A	
96	HQ184190	K3	Felis catus	South Korea	2008	A	
97	HQ184191	K49	Felis catus	South Korea	2008	A	
98	HQ184195	K49	Felis catus	South Korea	2008	S	
99	HQ184196	K50	Felis catus	South Korea	2008	A	
100	HQ184192	K7	Felis catus	South Korea	2008	A	
101	HQ184197	KS11	Felis catus	South Korea	2008	A	
102	HQ184198	KS18	Felis catus	South Korea	2008	A	
103	HQ184204	KS2	Felis catus	South Korea	2008	A	
104	HQ184199	KS23	Felis catus	South Korea	2008	A	
105	HQ184200	KS42	Felis catus	South Korea	2008	A	
106	HQ184201	KS45	Felis catus	South Korea	2008	A	
107	HQ184202	KS47	Felis catus	South Korea	2008	A	
108	HQ184203	KS58	Felis catus	South Korea	2008	A	
109	KT240130	PT005/08	Felis catus	Portugal	2008	A	
110	KU248463	PT015/08	Felis catus	Portugal	2008	A	
111	KT240131	PT022/08	Felis catus	Portugal	2008	A	
112	EU498680	Purevax-Merial vaccine	Felis catus	Italy	2008	A	
113	JF422105	PT09	Egyptian mongoose	Portugal	2009	A	
114	JX475259	CO/545/10	Puma concolor	USA	2010	A	
115	JX475253	CO/546/10	Puma concolor	USA	2010	T	
116	JX475245	CO/952/10	Puma concolor	USA	2010	A	
117	JX475254	CO/977/10	Puma concolor	USA	2010	A	0/7
118	OR566998	RJ10182010	Felis catus	Brazil	2010	A	
119	JN867593	CA/208-A/10	Procyon lotor	USA	2010	A	
120	JX411926	PT10-newCPV-2b	Stone marten	Portugal	2010	A	
121	JX475256	CO/1103/11	Puma concolor	USA	2011	A	
122	OR567000	RJ10962011	Felis catus	Brazil	2011	A	0/4
123	JX048608	FPV-1	Felis catus	China/Taiwan	2011	A	
124	OR566999	RJ10852011	Felis catus	Brazil	2011	A	
125	JX475270	GA/1/12	Procyon lotor	USA	2012	A	3/8
126	OR567001	RJ11592012	Felis catus	Brazil	2012	S	(37.5%)

127	OR567002	RJ11602012	Felis catus	Brazil	2012	S	
128	OR567003	RJ11612012	Felis catus	Brazil	2012	S	
129	KC473946	GD1209YGP	Felis catus	China/Guangzhou	2012	A	
130	KJ813895	MA/188	Procyon lotor	USA	2012	A	
131	KJ813894	MA/190	Raccoon	USA	2012	A	
132	KT240132	PT183/12	Felis catus	Portugal	2012	A	
133	KT240134	PT210/13	Felis catus	Portugal	2013	A	1/5 (20%)
134	OR567004	RJ11892013	Felis catus	Brazil	2013	A	
135	OR567005	RJ11982013	Felis catus	Brazil	2013	S	
136	KJ813893	Bobcat/ND/979	Lynx rufus	USA	2013	A	
137	KT240133	PT083/13	Felis catus	Portugal	2013	A	
138	OP985508	C1	Felis catus	Nigeria	2014	A	0/18
139	OP985509	C2	Felis catus	Nigeria	2014	A	
140	OP985510	C3	Felis catus	Nigeria	2014	A	
141	OP985512	C10	Felis catus	Nigeria	2014	A	
142	OP985513	C14	Felis catus	Nigeria	2014	A	
143	OP985514	C18	Felis catus	Nigeria	2014	A	
144	OP985515	C32	Felis catus	Nigeria	2014	A	
145	OP985516	C39	Felis catus	Nigeria	2014	A	
146	OP985511	C5	Felis catus	Nigeria	2014	A	
147	OP985517	C51	Felis catus	Nigeria	2014	A	
148	OP985518	C58	Felis catus	Nigeria	2014	A	
149	OP985519	C64	Felis catus	Nigeria	2014	A	
150	OP985520	C71	Felis catus	Nigeria	2014	A	
151	OP985521	C78	Felis catus	Nigeria	2014	A	
152	OP985522	C92	Felis catus	Nigeria	2014	A	
153	KT240135	PT264/14	Felis catus	Portugal	2014	A	
154	KU248464	PT265/14	Felis catus	Portugal	2014	A	
155	KT240136	PT271/14	Felis catus	Portugal	2014	A	
156	MH669800	VT01	Prionodon linsang	Thailand	2015	A	
157	MK671151	16CC1106	Felis catus	China/Jilin	2016	A	0/16
158	MK671154	16JZ0601	Felis catus	China/Liaoning	2016	A	

159	MK671155	16SY0601	Felis catus	China/Liaoning	2016	A	
160	MF541121	CC-02/16	Felis catus	China/Jilin	2016	A	
161	MH329286	F2016019	Felis catus	China/Henan	2016	A	
162	MK671150	16CC0806	Felis catus	China/Jilin	2016	A	
163	MK671152	16JL0804	Felis catus	China/Jilin	2016	A	
164	MK671153	16JL1205	Felis catus	China/Jilin	2016	A	
165	MK671156	16SY0711	Felis catus	China/Liaoning	2016	A	
166	MF541119	BC-02/16	Felis catus	China/Jilin	2016	A	
167	MF541120	BJ-03/16	Felis catus	China/Beijing	2016	A	
168	MF541122	HRB-01/16	Felis catus	China/Heilongjiang	2016	A	
169	MF541123	JL-01/17-03	Felis catus	China/Jilin	2016	A	
170	MF541124	JL-03/17-05	Felis catus	China/Jilin	2016	A	
171	MF541125	JL-04/16	Felis catus	China/Jilin	2016	A	
172	MF541140	SP-01/16	Felis catus	China/Jilin	2016	A	
173	MK671157	17BC0704	Felis catus	China/Jilin	2017	A	
174	MK671159	17CC0308	Felis catus	China/Jilin	2017	S	
175	MK671160	17DD0501	Felis catus	China/Liaoning	2017	A	
176	MK671168	17SY0302	Felis catus	China/Liaoning	2017	A	
177	MK671169	17SY0402	Felis catus	China/Liaoning	2017	S	
178	MT274377	51 FOX	Red fox	Italy	2017	T	
179	MK982094	CHJL-Siberian Tiger-01	Tiger	China/Jilin	2017	A	
180	MK266782	Haerbin-05	Felis catus	China/Heilongjiang	2017	A	
181	MF541129	JL-12/17-05	Felis catus	China/Jilin	2017	A	5/49
182	MF541132	JL-24/17-05	Felis catus	China/Jilin	2017	A	10%
183	MF541134	JL-29/17-05	Felis catus	China/Jilin	2017	A	
184	MK295775	JL-3	Felis catus	China/Jilin	2017	A	
185	MZ442312	SMU-D3	Felis catus	China/Sichuan	2017	S	
186	MK671158	17BC0801	Felis catus	China/Jilin	2017	A	
187	OP153925	17D01	Felis catus	South Korea	2017	A	
188	OP153926	17D02	Felis catus	South Korea	2017	A	
189	MK671161	17DD0902	Felis catus	China/Liaoning	2017	A	
190	MK671162	17HRB0505	Felis catus	China/Heilongjiang	2017	A	

191	MK671163	17HRB1001	Felis catus	China/Heilongjiang	2017	A
192	MK671164	17JL0704	Felis catus	China/Jilin	2017	S
193	MK671165	17JLSY0701	Felis catus	China/Jilin	2017	A
194	MK671166	17JLSY0901	Felis catus	China/Jilin	2017	A
195	MK671167	17SP0503	Felis catus	China/Jilin	2017	A
196	MK671170	17SY0503	Felis catus	China/Liaoning	2017	A
197	MK671171	17SY0902	Felis catus	China/Liaoning	2017	A
198	MK266791	Chengdu-01	Felis catus	China/Sichuan	2017	A
199	MK266790	Chengdu-03	Felis catus	China/Sichuan	2017	A
200	MK266788	Guiyang01	Felis catus	China/Guizhou	2017	A
201	MK266789	Guiyang01	Felis catus	China/Guizhou	2017	A
202	MK266784	Haerbin-13	Felis catus	China/Heilongjiang	2017	A
203	MK357739	HN3	Canis lupus familiaris	Vietnam	2017	A
204	MZ508523	17DIAPD55048/2	Felis catus	Italy	2017	S
205	MF541126	JL-04/17-03	Felis catus	China/Jilin	2017	A
206	MF541127	JL-07/17-05	Felis catus	China/Jilin	2017	A
207	MF541128	JL-10/17-06	Felis catus	China/Jilin	2017	A
208	MF541130	JL-19/17-06	Felis catus	China/Jilin	2017	A
209	MF541131	JL-20/17-05	Felis catus	China/Jilin	2017	A
210	MF541133	JL-28/17-05	Felis catus	China/Jilin	2017	A
211	MF541135	JL-33/17-05	Felis catus	China/Jilin	2017	A
212	MF541136	JL-34/17-05	Felis catus	China/Jilin	2017	A
213	MF541137	JL-40/17-05	Felis catus	China/Jilin	2017	A
214	MF541138	JL-47/17-05	Felis catus	China/Jilin	2017	A
215	MF541139	JT-01/17-03	Felis catus	China/Jilin	2017	A
216	MK266792	Shenyang-01	Felis catus	China/Liaoning	2017	A
217	MK266798	Shenyang-05	Felis catus	China/Liaoning	2017	A
218	MK266786	Shenyang-19	Felis catus	China/Liaoning	2017	A
219	MK266787	Shenyang-41	Felis catus	China/Liaoning	2017	A
220	MK266785	Shenyang-5	Felis catus	China/Liaoning	2017	A
221	MZ442309	SMU-D4	Felis catus	China/Sichuan	2017	A
222	MK671172	18BC0504	Felis catus	China/Jilin	2018	A

223	MK671173	18CC0102	Felis catus	China/Jilin	2018	A	21%
224	MK671177	18HRB0102	Felis catus	China/Heilongjiang	2018	A	
225	MK671178	18HRB0801	Felis catus	China/Heilongjiang	2018	A	
226	MK671181	18JL0602	Felis catus	China/Jilin	2018	A	
227	MW091486	CD-2	Giant panda	China/Sichuan	2018	A	
228	ON646201	DL01	Felis catus	China/Liaoning	2018	A	
229	ON646202	DL02	Felis catus	China/Liaoning	2018	A	
230	ON646203	DL03	Felis catus	China/Liaoning	2018	A	
231	ON646204	DL04	Felis catus	China/Liaoning	2018	A	
232	ON646205	DL05	Felis catus	China/Liaoning	2018	S	
233	ON646206	DL06	Felis catus	China/Liaoning	2018	A	
234	ON646207	DL07	Felis catus	China/Liaoning	2018	A	
235	MK357738	HN39AA	Canis lupus familiaris	Viet Nam	2018	A	
236	MK301396	QDDX	Felis catus	China/Shandong	2018	S	
237	MZ442307	SMU-D46	Felis catus	China/Sichuan	2018	A	
238	MZ442306	SMU-D50	Felis catus	China/Sichuan	2018	A	
239	MK671174	18CC0718	Felis catus	China/Jilin	2018	A	
240	MK671175	18CC0909	Felis catus	China/Jilin	2018	S	
241	OP153927	18D01	Felis catus	South Korea	2018	A	
242	MK671176	18DD0302	Felis catus	China/Liaoning	2018	A	
243	MK671179	18HRB1002	Felis catus	China/Heilongjiang	2018	S	
244	MK671180	18JL0105	Felis catus	China/Jilin	2018	A	
245	MK671182	18JZ0501	Felis catus	China/Liaoning	2018	A	
246	MK671183	18LY0701	Felis catus	China/Liaoning	2018	A	
247	MK671184	18LY0801	Felis catus	China/Liaoning	2018	S	
248	MK671185	18LY0902	Felis catus	China/Liaoning	2018	S	
249	MK671186	18QQHE0503	Felis catus	China/Heilongjiang	2018	A	
250	MK671187	18SP0701	Felis catus	China/Jilin	2018	A	
251	MK671188	18SY0102	Felis catus	China/Liaoning	2018	A	
252	MK266797	Beijing-01	Felis catus	China/Beijing	2018	S	
253	MK266795	Beijing-L3	Felis catus	China/Beijing	2018	A	
254	MK266796	Beijing-L4	Felis catus	China/Beijing	2018	A	

255	MZ322607	CD2018	Giant panda	China/Sichuan	2018	A
256	MT857283	F1	Felis catus	Viet Nam	2018	A
257	MT857284	F2	Felis catus	Viet Nam	2018	A
258	MT857285	F3	Felis catus	Viet Nam	2018	A
259	MT857286	F4	Felis catus	Viet Nam	2018	A
260	MT857268	F5	Felis catus	Viet Nam	2018	A
261	MT857269	F6	Felis catus	Viet Nam	2018	A
262	MT857270	F7	Felis catus	Viet Nam	2018	A
263	MT857271	F8	Felis catus	Viet Nam	2018	A
264	MT857272	F9	Felis catus	Viet Nam	2018	A
265	MK266783	Haerbin-01	Felis catus	China/Heilongjiang	2018	A
266	MK357741	HN10	Canis lupus familiaris	Viet Nam	2018	A
267	OQ868533	HN1801	Felis catus	China/Henan	2018	A
268	OQ868534	HN1802	Felis catus	China/Henan	2018	S
269	OQ868535	HN1803	Felis catus	China/Henan	2018	S
270	OQ868536	HN1804	Felis catus	China/Henan	2018	S
271	OQ868537	HN1805	Felis catus	China/Henan	2018	S
272	OQ868538	HN1806	Felis catus	China/Henan	2018	A
273	MK357740	HN40AA	Canis lupus familiaris	Viet Nam	2018	A
274	MK357742	HN41AA	Canis lupus familiaris	Viet Nam	2018	A
275	MK357743	HN7	Canis lupus familiaris	Viet Nam	2018	A
276	MK266799	Jilin52	Felis catus	China/Jilin	2018	A
277	OR260993	PSY01-2	Felis catus	China/Beijing	2018	A
278	MZ442308	SMU-D14	Felis catus	China/Sichuan	2018	A
279	MZ442310	SMU-D18	Felis catus	China/Sichuan	2018	A
280	MZ442311	SMU-D28	Felis catus	China/Sichuan	2018	A
281	MZ442305	SMU-D53	Felis catus	China/Sichuan	2018	A
282	MK266793	Tanjin-01	Felis catus	China/Tianjin	2018	A
283	MK266794	Tianjin-02	Felis catus	China/Tianjin	2018	A
284	MN270937	VT2020	Canine	Thailand	2018	A
285	MZ508522	18DIAPD52063/2	Felis catus	Italy	2018	S
286	MZ508525	18DIAPD932/2	Felis catus	Italy	2018	S

287	OQ815872	FPV-15	Felis catus	China/Jiangsu	2018	S	
288	MT274378	245-1478	Badger	Italy	2019	A	
289	OQ868553	AH1904	Felis catus	China/Anhui	2019	S	
290	MT078767	BCC1	Felis catus	India	2019	A	
291	MT270584	BJ016	Felis catus	China/Beijing	2019	A	
292	MT270581	BJ050	Felis catus	China/Beijing	2019	S	
293	MT270580	BJ051	Felis catus	China/Beijing	2019	S	
294	MT270579	BJ058	Felis catus	China/Beijing	2019	S	
295	MT270578	BJ061	Felis catus	China/Beijing	2019	A	
296	MT270576	BJ090	Felis catus	China/Beijing	2019	S	
297	MT270574	BJ128	Felis catus	China/Beijing	2019	S	
298	MT270571	BJ240	Felis catus	China/Beijing	2019	S	
299	MT270567	BJ318	Felis catus	China/Beijing	2019	A	
300	MT270566	BJ319	Felis catus	China/Beijing	2019	A	
301	MT270564	BJ372	Felis catus	China/Beijing	2019	A	
302	MT270563	BJ379	Felis catus	China/Beijing	2019	A	
303	MT270561	BJ405	Felis catus	China/Beijing	2019	S	55/123<br>45%
304	MT270557	BJ461	Felis catus	China/Beijing	2019	S	
305	MT270556	BJ481	Felis catus	China/Beijing	2019	S	
306	MT270554	BJ502	Felis catus	China/Beijing	2019	S	
307	MT270553	BJ540	Felis catus	China/Beijing	2019	S	
308	MT270550	BJ557	Felis catus	China/Beijing	2019	S	
309	MT270548	BJ565	Felis catus	China/Beijing	2019	S	
310	MT270541	BJ619	Felis catus	China/Beijing	2019	S	
311	MT270540	BJ624	Felis catus	China/Beijing	2019	S	
312	MT270531	BJ728	Felis catus	China/Beijing	2019	S	
313	ON646208	DL08	Felis catus	China/Liaoning	2019	S	
314	ON646209	DL09	Felis catus	China/Liaoning	2019	S	
315	ON646210	DL12	Felis catus	China/Liaoning	2019	A	
316	ON646211	DL13	Felis catus	China/Liaoning	2019	A	
317	ON646212	DL14	Felis catus	China/Liaoning	2019	A	
318	ON646213	DL15	Felis catus	China/Liaoning	2019	A	

319	ON646214	DL16	Felis catus	China/Liaoning	2019	A	
320	ON646215	DL17	Felis catus	China/Liaoning	2019	A	
321	ON646216	DL18	Felis catus	China/Liaoning	2019	A	
322	ON646217	DL19	Felis catus	China/Liaoning	2019	A	
323	ON646218	DL20	Felis catus	China/Liaoning	2019	A	
324	MN418997	DLC01	Felis catus	China/Liaoning	2019	A	
325	MN418998	DLC02	Felis catus	China/Liaoning	2019	A	
326	MN418999	DLC03	Felis catus	China/Liaoning	2019	S	
327	MN419000	DLC04	Felis catus	China/Liaoning	2019	A	
328	MN419001	DLC05	Felis catus	China/Liaoning	2019	A	
329	MN419002	DLC06	Felis catus	China/Liaoning	2019	A	
330	MN419003	DLC07	Felis catus	China/Liaoning	2019	A	
331	MN419004	DLC08	Felis catus	China/Liaoning	2019	A	
332	OP471918	HBSJZ19-01	Felis catus	China/Hebei	2019	S	
333	OQ868539	HN1901	Felis catus	China/Henan	2019	S	
334	OQ868540	HN1902	Felis catus	China/Henan	2019	S	
335	OQ868541	HN1903	Felis catus	China/Henan	2019	S	
336	OQ868542	HN1904	Felis catus	China/Henan	2019	S	
337	OQ868543	HN1905	Felis catus	China/Henan	2019	S	
338	OQ868544	HN1906	Felis catus	China/Henan	2019	A	
339	OQ868545	HN1908	Felis catus	China/Henan	2019	A	
340	OQ868546	HN1909	Felis catus	China/Henan	2019	A	
341	OQ868547	HN1910	Felis catus	China/Henan	2019	S	
342	MT892650	SX	Felis catus	China/Shaanxi	2019	S	
343	OP153928	19D01	Felis catus	South Korea	2019	A	
344	OP153929	19D02	Felis catus	South Korea	2019	A	
345	OP153930	19D03	Felis catus	South Korea	2019	A	
346	OP153931	19D04	Felis catus	South Korea	2019	A	
347	OP153932	19D05	Felis catus	South Korea	2019	A	
348	OQ868550	AH1901	Felis catus	China/Anhui	2019	S	
349	OQ868551	AH1902	Felis catus	China/Anhui	2019	S	
350	OQ868552	AH1903	Felis catus	China/Anhui	2019	S	

351	MT078768	BCC13	Felis catus	India	2019	A	
352	MT270585	BJ006	Felis catus	China/Beijing	2019	A	
353	MT270583	BJ020	Felis catus	China/Beijing	2019	A	
354	MT270582	BJ025	Felis catus	China/Beijing	2019	A	
355	MT270577	BJ078	Felis catus	China/Beijing	2019	A	
356	MT270575	BJ094	Felis catus	China/Beijing	2019	S	
357	MT270573	BJ133	Felis catus	China/Beijing	2019	S	
358	MT270572	BJ235	Felis catus	China/Beijing	2019	S	
359	MT270570	BJ277	Felis catus	China/Beijing	2019	A	
360	MT270569	BJ308	Felis catus	China/Beijing	2019	S	
361	MT270568	BJ309	Felis catus	China/Beijing	2019	S	
362	MT270565	BJ338	Felis catus	China/Beijing	2019	S	
363	MT270562	BJ396	Felis catus	China/Beijing	2019	S	
364	MT270560	BJ416	Felis catus	China/Beijing	2019	S	
365	MT270559	BJ435	Felis catus	China/Beijing	2019	S	
366	MT270558	BJ440	Felis catus	China/Beijing	2019	S	
367	MT270555	BJ501	Felis catus	China/Beijing	2019	S	
368	MT270552	BJ552	Felis catus	China/Beijing	2019	S	
369	MT270551	BJ554	Felis catus	China/Beijing	2019	S	
370	MT270549	BJ562	Felis catus	China/Beijing	2019	A	
371	MT270547	BJ572	Felis catus	China/Beijing	2019	S	
372	MT270546	BJ574	Felis catus	China/Beijing	2019	A	
373	MT270545	BJ577	Felis catus	China/Beijing	2019	A	
374	MT270544	BJ582	Felis catus	China/Beijing	2019	S	
375	MT270543	BJ588	Felis catus	China/Beijing	2019	A	
376	MT270542	BJ594	Felis catus	China/Beijing	2019	S	
377	MT270539	BJ625	Felis catus	China/Beijing	2019	S	
378	MT270538	BJ629	Felis catus	China/Beijing	2019	S	
379	MT270537	BJ638	Felis catus	China/Beijing	2019	S	
380	MT270536	BJ644	Felis catus	China/Beijing	2019	S	
381	OP471917	CC19-02	Felis catus	China/Jilin	2019	S	
382	MT078769	CPF6	Felis catus	India	2019	A	

383	MT078770	CV3	Felis catus	India	2019	A	
384	MN419007	DLC10	Felis catus	China/Liaoning	2019	A	
385	MN419008	DLC11	Felis catus	China/Liaoning	2019	A	
386	MN419009	DLC12	Felis catus	China/Liaoning	2019	A	
387	MN419010	DLC13	Felis catus	China/Liaoning	2019	A	
388	MN419011	DLC14	Felis catus	China/Liaoning	2019	A	
389	MN419012	DLC15	Felis catus	China/Liaoning	2019	S	
390	MN419013	DLC16	Felis catus	China/Liaoning	2019	A	
391	MN419005	DLC91	Felis catus	China/Liaoning	2019	A	
392	MN419006	DLC92	Felis catus	China/Liaoning	2019	A	
393	OM937916	EGY39-566	Felis catus	Egypt	2019	A	
394	MT857273	F10	Felis catus	Viet Nam	2019	A	
395	MT857274	F11	Felis catus	Viet Nam	2019	A	
396	MT857275	F12	Felis catus	Viet Nam	2019	A	
397	MT857276	F13	Felis catus	Viet Nam	2019	A	
398	MT857277	F14	Felis catus	Viet Nam	2019	A	
399	MT857278	F15	Felis catus	Viet Nam	2019	A	
400	MT857279	F16	Felis catus	Viet Nam	2019	A	
401	MT857280	F17	Felis catus	Viet Nam	2019	A	
402	MT857281	F18	Felis catus	Viet Nam	2019	A	
403	MT857282	F19	Felis catus	Viet Nam	2019	A	
404	OP471919	HBSJZ19-02	Felis catus	China/Hebei	2019	A	
405	MZ836347	JN-11	Felis catus	China/Shandong	2019	S	
406	MZ836378	JN-12	Felis catus	China/Shandong	2019	A	
407	MW017596	JSYZ-85	Canine	China/Jiangsu	2019	S	
408	MT078771	PDC3	Felis catus	India	2019	A	
409	MZ442304	SMU-D74	Felis catus	China/Sichuan	2019	A	
410	MZ442303	SMU-D87	Felis catus	China/Sichuan	2019	A	
411	OQ815870	FPV-6	Felis catus	China/Hubei	2020	S	
412	OQ868554	AH2001	Felis catus	China/Anhui	2020	S	59/86<br>67%
413	OQ868557	AH2004	Felis catus	China/Anhui	2020	S	
414	OQ868559	AH2007	Felis catus	China/Anhui	2020	S	

415	MT270532	BJ700	Felis catus	China/Beijing	2020	S	
416	MZ836377	JN-87	Felis catus	China/Shandong	2020	A	
417	MZ836376	JN-90	Felis catus	China/Shandong	2020	A	
418	MZ836375	JN-91	Felis catus	China/Shandong	2020	A	
419	MZ836374	JN-92	Felis catus	China/Shandong	2020	A	
420	OQ868562	JS2001	Felis catus	China/Jiangsu	2020	S	
421	MW017628	JSYZ-122	Felis catus	China/Jiangsu	2020	A	
422	MW017629	JSYZ-123	Felis catus	China/Jiangsu	2020	S	
423	MW017630	JSYZ-124	Felis catus	China/Jiangsu	2020	S	
424	MZ391097	kucuk	Felis catus	Turkey	2020	A	
425	OR551217	Luoyang-01	Felis catus	China/Henan	2020	S	
426	OR551218	Luoyang-03	Felis catus	China/Henan	2020	S	
427	MW017625	SH-118	Felis catus	China/Shanghai	2020	A	
428	MW017626	SH-120	Felis catus	China/Shanghai	2020	S	
429	MZ836370	TZ-104	Felis catus	China/Beijing	2020	A	
430	MZ836369	TZ-108	Felis catus	China/Beijing	2020	A	
431	MZ836368	TZ-112	Felis catus	China/Beijing	2020	S	
432	MZ836366	TZ-122	Felis catus	China/Beijing	2020	A	
433	MZ836365	TZ-124	Felis catus	China/Beijing	2020	A	
434	MZ836364	TZ-133	Felis catus	China/Beijing	2020	S	
435	MZ836363	TZ-135	Felis catus	China/Beijing	2020	S	
436	MZ836362	TZ-148	Felis catus	China/Beijing	2020	A	
437	MZ836361	TZ-185	Felis catus	China/Beijing	2020	S	
438	MZ836360	TZ-193	Felis catus	China/Beijing	2020	A	
439	MZ836358	TZ-235	Felis catus	China/Beijing	2020	S	
440	MZ836357	TZ-236	Felis catus	China/Beijing	2020	A	
441	MZ836356	TZ-237	Felis catus	China/Beijing	2020	S	
442	MZ836355	TZ-238	Felis catus	China/Beijing	2020	S	
443	MZ836354	TZ-239	Felis catus	China/Beijing	2020	S	
444	MZ836353	TZ-240	Felis catus	China/Beijing	2020	S	
445	MZ836352	TZ-241	Felis catus	China/Beijing	2020	S	
446	MZ836351	TZ-242	Felis catus	China/Beijing	2020	A	

447	MW495835	ZJFPV10	Felis catus	China/Zhejiang	2020	A	
448	MW495836	ZJFPV11	Felis catus	China/Zhejiang	2020	S	
449	MW495837	ZJFPV12	Felis catus	China/Zhejiang	2020	S	
450	MW495838	ZJFPV13	Felis catus	China/Zhejiang	2020	A	
451	MW495839	ZJFPV14	Felis catus	China/Zhejiang	2020	S	
452	MW495840	ZJFPV15	Felis catus	China/Zhejiang	2020	S	
453	MW495841	ZJFPV16	Felis catus	China/Zhejiang	2020	S	
454	MW495842	ZJFPV17	Felis catus	China/Zhejiang	2020	S	
455	MW495843	ZJFPV18	Felis catus	China/Zhejiang	2020	S	
456	MW495844	ZJFPV19	Felis catus	China/Zhejiang	2020	S	
457	MW495829	ZJFPV2	Felis catus	China/Zhejiang	2020	S	
458	MW495845	ZJFPV20	Felis catus	China/Zhejiang	2020	S	
459	MW495846	ZJFPV21	Felis catus	China/Zhejiang	2020	S	
460	MW495847	ZJFPV22	Felis catus	China/Zhejiang	2020	S	
461	MW495848	ZJFPV23	Felis catus	China/Zhejiang	2020	A	
462	MW495830	ZJFPV4	Felis catus	China/Zhejiang	2020	A	
463	MW495831	ZJFPV5	Felis catus	China/Zhejiang	2020	A	
464	MW495832	ZJFPV6	Felis catus	China/Zhejiang	2020	S	
465	MW495833	ZJFPV7	Felis catus	China/Zhejiang	2020	A	
466	MW495834	ZJFPV8	Felis catus	China/Zhejiang	2020	A	
467	MW017616	ZJHN-135	Canine	China/Zhejiang	2020	S	
468	MW017618	ZJHN-138	Canine	China/Zhejiang	2020	S	
469	OQ868555	AH2002	Felis catus	China/Anhui	2020	S	
470	OQ868556	AH2003	Felis catus	China/Anhui	2020	S	
471	OQ868558	AH2006	Felis catus	China/Anhui	2020	A	
472	OQ868560	AH2009	Felis catus	China/Anhui	2020	S	
473	OQ868563	AH2010	Felis catus	China/Anhui	2020	S	
474	OQ868561	AH2011	Felis catus	China/Anhui	2020	S	
475	OQ868564	AH2012	Felis catus	China/Anhui	2020	S	
476	MZ391096	Barut	Felis catus	Turkey	2020	A	
477	OQ868548	HB2001	Felis catus	China/Hubei	2020	S	
478	OQ868549	HB2002	Felis catus	China/Hubei	2020	S	

479	OQ868569	HB2003	Felis catus	China/Hubei	2020	S	
480	MZ836373	JN-96	Felis catus	China/Shandong	2020	S	
481	OQ868565	JS2002	Felis catus	China/Jiangsu	2020	S	
482	MW791426	JSYZ-168	Felis catus	China/Jiangsu	2020	S	
483	MW791427	JSYZ-169	Felis catus	China/Jiangsu	2020	S	
484	OR551219	Luoyang-08	Felis catus	China/Henan	2020	S	
485	OR551220	Luoyang-13	Felis catus	China/Henan	2020	A	
486	OR551221	Luoyang-19	Felis catus	China/Henan	2020	S	
487	MW017627	SH-121	Felis catus	China/Shanghai	2020	A	
488	MZ442302	SMU-F33	Felis catus	China/Sichuan	2020	S	
489	MZ442313	SMU-SC20-2	Felis catus	China/Sichuan	2020	S	
490	MZ442314	SMU-SC20-6	Felis catus	China/Sichuan	2020	S	
491	MZ836359	TZ-195	Felis catus	China/Beijing	2020	A	
492	MZ836350	TZ-243	Felis catus	China/Beijing	2020	S	
493	MZ836371	TZ-99	Felis catus	China/Beijing	2020	A	
494	OR551222	Zhengzhou-01	Felis catus	China/Henan	2020	S	
495	OR551224	Zhengzhou-11	Felis catus	China/Henan	2020	S	
496	MW017631	ZJHN-126	Felis catus	China/Zhejiang	2020	S	
497	PP738170	Cat-1	Felis catus	China/Henan	2021	A	
498	PP738171	Cat-2	Felis catus	China/Henan	2021	S	
499	PP738172	Cat-3	Felis catus	China/Henan	2021	S	
500	PP738173	Cat-4	Felis catus	China/Henan	2021	A	
501	PP738174	Cat-5	Felis catus	China/Henan	2021	A	
502	ON185552	1724-HU	Felis catus	Hungary	2021	A	
503	OQ718429	IT	Felis catus	Italy	2021	A	60/89
504	OR227624	CH/YCYH/2021	Felis catus	China/Jiangxi	2021	S	67%
505	OR551227	Anyang-02	Felis catus	China/Henan	2021	S	
506	MT270533	BJ698	Felis catus	China/Beijing	2021	S	
507	OQ398386	FPV003	Felis catus	China/Shandong	2021	A	
508	OQ398387	FPV008	Felis catus	China/Shandong	2021	A	
509	OQ398388	FPV013	Felis catus	China/Shandong	2021	A	
510	OQ398389	FPV014	Felis catus	China/Shandong	2021	A	

511	OQ398390	FPV021	Felis catus	China/Shandong	2021	A
512	OQ398391	FPV026	Felis catus	China/Shandong	2021	A
513	OQ398392	FPV027	Felis catus	China/Shandong	2021	A
514	OQ398394	FPV029	Felis catus	China/Shandong	2021	S
515	OQ398395	FPV030	Felis catus	China/Shandong	2021	S
516	OQ398396	FPV036	Felis catus	China/Shandong	2021	S
517	OQ398398	FPV038	Felis catus	China/Shandong	2021	S
518	OQ398399	FPV039	Felis catus	China/Shandong	2021	S
519	OQ398401	FPV041	Felis catus	China/Shandong	2021	S
520	OQ398402	FPV042	Felis catus	China/Shandong	2021	S
521	OQ398403	FPV043	Felis catus	China/Shandong	2021	S
522	OQ398418	FPV046	Felis catus	China/Shandong	2021	A
523	OQ398419	FPV048	Felis catus	China/Shandong	2021	A
524	OQ398406	FPV072	Felis catus	China/Shandong	2021	S
525	OQ398408	FPV077	Felis catus	China/Shandong	2021	S
526	OQ398409	FPV080	Felis catus	China/Shandong	2021	S
527	OQ398413	FPV086	Felis catus	China/Shandong	2021	S
528	OP796708	JSNJ-21G4	Felis catus	China/Jiangsu	2021	S
529	OP796709	JSNJ-21G5	Felis catus	China/Jiangsu	2021	S
530	OP796706	SH-21D2	Felis catus	China/Shanghai	2021	S
531	OQ615261	UFUUSP15	Felis catus	Brazil	2021	A
532	OQ615262	UFUUSP16	Felis catus	Brazil	2021	A
533	OQ615263	UFUUSP17	Felis catus	Brazil	2021	A
534	OM885379	Yanji10	Felis catus	China/Jilin	2021	S
535	OM885380	Yanji11	Felis catus	China/Jilin	2021	S
536	OM885381	Yanji12	Felis catus	China/Jilin	2021	A
537	OM885382	Yanji13	Felis catus	China/Jilin	2021	S
538	OM885383	Yanji15	Felis catus	China/Jilin	2021	S
539	OM885384	Yanji18	Felis catus	China/Jilin	2021	S
540	OM918770	Yanji23	Felis catus	China/Jilin	2021	A
541	OM918780	Yanji35	Felis catus	China/Jilin	2021	S
542	OM885377	Yanji8	Felis catus	China/Jilin	2021	L

543	OM885378	Yanji9	Felis catus	China/Jilin	2021	S
544	OQ398393	FPV028	Felis catus	China/Shandong	2021	A
545	OQ398397	FPV037	Felis catus	China/Shandong	2021	S
546	OQ398400	FPV040	Felis catus	China/Shandong	2021	S
547	OQ398404	FPV044	Felis catus	China/Shandong	2021	S
548	OQ398405	FPV045	Felis catus	China/Shandong	2021	S
549	OQ398420	FPV049	Felis catus	China/Shandong	2021	A
550	OQ398421	FPV050	Felis catus	China/Shandong	2021	A
551	OQ398422	FPV055	Felis catus	China/Shandong	2021	S
552	OQ398407	FPV076	Felis catus	China/Shandong	2021	S
553	OQ398410	FPV081	Felis catus	China/Shandong	2021	S
554	OQ398411	FPV082	Felis catus	China/Shandong	2021	S
555	OQ398412	FPV085	Felis catus	China/Shandong	2021	S
556	OQ398414	FPV087	Felis catus	China/Shandong	2021	S
557	OQ398415	FPV088	Felis catus	China/Shandong	2021	S
558	OQ398416	FPV089	Felis catus	China/Shandong	2021	S
559	OQ398417	FPV090	Felis catus	China/Shandong	2021	S
560	OQ868566	HN2101	Felis catus	China/Henan	2021	S
561	OQ868567	HN2105	Felis catus	China/Henan	2021	S
562	OR194141	SDYT2	Felis catus	China/Shandong	2021	S
563	OP796707	SH-21D4	Felis catus	China/Shanghai	2021	S
564	OM212011	Yanji	Felis catus	China/Jilin	2021	S
565	OM918783	Yanji17	Felis catus	China/Jilin	2021	S
566	OM322821	Yanji2	Felis catus	China/Jilin	2021	A
567	OM918771	Yanji24	Felis catus	China/Jilin	2021	S
568	OM918772	Yanji25	Felis catus	China/Jilin	2021	S
569	OM918784	Yanji26	Felis catus	China/Jilin	2021	S
570	OM918773	Yanji27	Felis catus	China/Jilin	2021	A
571	OM918774	Yanji28	Felis catus	China/Jilin	2021	S
572	OM918775	Yanji29	Felis catus	China/Jilin	2021	S
573	OM885373	Yanji3	Felis catus	China/Jilin	2021	S
574	OM918776	Yanji30	Felis catus	China/Jilin	2021	L

575	OM918777	Yanji31	Felis catus	China/Jilin	2021	S	
576	OM918778	Yanji32	Felis catus	China/Jilin	2021	S	
577	OM918779	Yanji33	Felis catus	China/Jilin	2021	S	
578	OM918781	Yanji36	Felis catus	China/Jilin	2021	A	
579	OM918785	Yanji37	Felis catus	China/Jilin	2021	S	
580	OM918782	Yanji38	Felis catus	China/Jilin	2021	S	
581	OM885374	Yanji4	Felis catus	China/Jilin	2021	S	
582	OM885375	Yanji5	Felis catus	China/Jilin	2021	A	
583	OM885376	Yanji6	Felis catus	China/Jilin	2021	S	
584	OR551223	Zhengzhou-07	Felis catus	China/Henan	2021	S	
585	OR551225	Zhengzhou-26	Felis catus	China/Henan	2021	A	
586	OR211672	AHWH55	Felis catus	China/Anhui	2022	A	
587	MT270534	BJ663	Felis catus	China/Beijing	2022	A	
588	OR399569	FPV109	Felis catus	China/Jiangsu	2022	S	
589	OR399570	FPV111	Felis catus	China/Jiangsu	2022	S	
590	OR399559	FPV13	Felis catus	China/Jiangsu	2022	S	
591	OR399571	FPV132	Felis catus	China/Jiangsu	2022	S	
592	OR399572	FPV133	Felis catus	China/Jiangsu	2022	S	
593	OR399573	FPV141	Felis catus	China/Jiangsu	2022	S	
594	OR399574	FPV143	Felis catus	China/Jiangsu	2022	S	
595	OR399575	FPV149	Felis catus	China/Jiangsu	2022	S	
596	OR399560	FPV19	Felis catus	China/Jiangsu	2022	S	75/87 86%
597	OR399565	FPV19	Felis catus	China/Jiangsu	2022	S	
598	OR399561	FPV34	Felis catus	China/Jiangsu	2022	S	
599	OR399562	FPV41	Felis catus	China/Jiangsu	2022	S	
600	OR399563	FPV42	Felis catus	China/Jiangsu	2022	S	
601	OR399564	FPV56	Felis catus	China/Jiangsu	2022	S	
602	OR399566	FPV73	Felis catus	China/Jiangsu	2022	S	
603	OR399567	FPV77	Felis catus	China/Jiangsu	2022	S	
604	OR399568	FPV89	Felis catus	China/Jiangsu	2022	S	
605	OQ868568	JS2201	Felis catus	China/Jiangsu	2022	S	
606	OR194110	JSZJ1	Felis catus	China/Jiangsu	2022	S	

607	OQ570642	JZ2022	Felis catus	China/Liaoning	2022	S
608	OQ535501	SDQD14	Felis catus	China/Shandong	2022	S
609	OR194121	SH1	Felis catus	China/Shanghai	2022	S
610	OR194124	SH4	Felis catus	China/Shanghai	2022	S
611	OR194125	SH5	Felis catus	China/Shanghai	2022	S
612	OP796714	ZJHN-2206	Felis catus	China/Zhejiang	2022	A
613	OP796716	ZJHN-2208	Felis catus	China/Zhejiang	2022	A
614	OR211675	AHWH52	Felis catus	China/Anhui	2022	S
615	OR211674	AHWH53	Felis catus	China/Anhui	2022	S
616	OR211673	AHWH54	Felis catus	China/Anhui	2022	S
617	OR194142	AHWH56	Felis catus	China/Anhui	2022	S
618	OR194133	FJFZ1	Felis catus	China/Fujian	2022	S
619	OR194134	FJFZ2	Felis catus	China/Fujian	2022	S
620	OR194135	FJFZ3	Felis catus	China/Fujian	2022	S
621	OR211676	FJFZ4	Felis catus	China/Fujian	2022	S
622	OR194129	GDGZ1	Felis catus	China/Guangdong	2022	S
623	OR194130	GDGZ2	Felis catus	China/Guangdong	2022	S
624	OR194131	GDGZ3	Felis catus	China/Guangdong	2022	S
625	OR194132	GDGZ4	Felis catus	China/Guangdong	2022	S
626	OR194126	HNZZ1	Felis catus	China/Henan	2022	S
627	OR194127	HNZZ2	Felis catus	China/Henan	2022	S
628	OR194128	HNZZ3	Felis catus	China/Henan	2022	S
629	OR194136	JSWX1	Felis catus	China/Jiangsu	2022	S
630	OR194137	JSWX2	Felis catus	China/Jiangsu	2022	S
631	OR194138	JSWX3	Felis catus	China/Jiangsu	2022	S
632	OR194139	JSWX4	Felis catus	China/Jiangsu	2022	S
633	OR194140	JSWX5	Felis catus	China/Jiangsu	2022	S
634	OR194111	JSZJ2	Felis catus	China/Jiangsu	2022	S
635	OQ869254	LZ05	Canine	China/Gansu	2022	S
636	OR783313	LZ092022	Felis catus	China/Gansu	2022	S
637	ON605652	RCP vaccine	Felis catus	Australia	2022	A
638	OQ535504	SDQD21	Felis catus	China/Shandong	2022	S

639	OQ535505	SDQD23	Felis catus	China/Shandong	2022	S
640	OQ535496	SDQD6	Felis catus	China/Shandong	2022	S
641	OQ535507	SDYT22	Felis catus	China/Shandong	2022	A
642	OR211671	SDYT3	Felis catus	China/Shandong	2022	A
643	OR194122	SH2	Felis catus	China/Shanghai	2022	S
644	OR194123	SH3	Felis catus	China/Shanghai	2022	S
645	OR551226	Xinxiang-05	Felis catus	China/Henan	2022	S
646	OP796713	ZJHN-2205	Felis catus	China/Zhejiang	2022	A
647	OP796715	ZJHN-2207	Felis catus	China/Zhejiang	2022	S
648	OP796710	ZJHZ-2202	Felis catus	China/Zhejiang	2022	S
649	OP796711	ZJHZ-2203	Felis catus	China/Zhejiang	2022	S
650	OP796712	ZJHZ-2204	Felis catus	China/Zhejiang	2022	S
651	OR399559	13	Felis catus	China/Jiangsu	2022	S
652	OR399560	19	Felis catus	China/Jiangsu	2022	S
653	OR399561	34	Felis catus	China/Jiangsu	2022	S
654	OR399562	41	Felis catus	China/Jiangsu	2022	S
655	OR399563	42	Felis catus	China/Jiangsu	2022	S
656	OR399564	56	Felis catus	China/Jiangsu	2022	S
657	OR399565	59	Felis catus	China/Jiangsu	2022	S
658	OR399566	73	Felis catus	China/Jiangsu	2022	S
659	OR399567	77	Felis catus	China/Jiangsu	2022	S
660	OR399568	89	Felis catus	China/Jiangsu	2022	S
661	OR709671	QD22-1	Felis catus	China/Shandong	2022	S
662	OR727315	QD22-5	Felis catus	China/Shandong	2022	S
663	OR727316	QD22-4	Felis catus	China/Shandong	2022	S
664	OR727317	QD22-8	Felis catus	China/Shandong	2022	A
665	OR727318	QD22-3	Felis catus	China/Shandong	2022	S
666	OR727319	QD22-13	Felis catus	China/Shandong	2022	S
667	PP035815	MZ26	Felis catus	India	2022	S
668	PP035816	MZ33	Felis catus	India	2022	S
669	PP035817	MZ35	Felis catus	India	2022	S
670	PP419033	MZ27	Felis catus	India	2022	A

671	PP419034	MZ29	Felis catus	India	2022	A	
672	PP419035	MZ30	Felis catus	India	2022	A	
673	OR365078	KTPV-2305	Siberian tiger	South Korea	2023	A	
674	MT270535	BJ662	Felis catus	China/Beijing	2023	S	
675	OQ615264	Nobivac vaccine strain	Felis catus	Brazil	2023	A	
676	PP781541	CTU-AG1	Felis catus	Viet Nam	2023	S	
677	PP781542	CTU-AG2	Felis catus	Viet Nam	2023	S	
678	PP781543	CTU-AG3	Felis catus	Viet Nam	2023	S	
679	PP781544	CTU-AG4	Felis catus	Viet Nam	2023	S	
680	PP781545	CTU-AG5	Felis catus	Viet Nam	2023	S	
681	PP781546	CTU-AG6	Felis catus	Viet Nam	2023	S	
682	PP781547	CTU-AG7	Felis catus	Viet Nam	2023	S	
683	PP781548	CTU-AG8	Felis catus	Viet Nam	2023	S	
684	PP781533	CTU-CM1	Felis catus	Viet Nam	2023	A	
685	PP781534	CTU-CM2	Felis catus	Viet Nam	2023	S	
686	PP781535	CTU-CM3	Felis catus	Viet Nam	2023	A	
687	PP781536	CTU-CM4	Felis catus	Viet Nam	2023	A	45/101<br>21/25, 84%, China<br>24/37, 65%, Viet Nam
688	PP781537	CTU-CM5	Felis catus	Viet Nam	2023	A	
689	PP781538	CTU-CM6	Felis catus	Viet Nam	2023	A	
690	PP781539	CTU-CM7	Felis catus	Viet Nam	2023	S	
691	PP781540	CTU-CM8	Felis catus	Viet Nam	2023	S	
692	PP663044	FVMCU 8	Felis catus	Egypt	2023	A	
693	PP663045	FVMCU (10)	Felis catus	Egypt	2023	A	
694	PP663046	FVMCU (12)	Felis catus	Egypt	2023	A	
695	PP663047	FVMCU (16)	Felis catus	Egypt	2023	A	
696	PP663048	FVMCU (17)	Felis catus	Egypt	2023	A	
697	PP663049	FVMCU (18)	Felis catus	Egypt	2023	A	
698	PP663050	FVMCU (20)	Felis catus	Egypt	2023	A	
699	PP663051	FVMCU (21)	Felis catus	Egypt	2023	A	
700	PP663052	FVMCU (22)	Felis catus	Egypt	2023	A	
701	PP663053	FVMCU (25)	Felis catus	Egypt	2023	A	
702	PP663054	FVMCU (26)	Felis catus	Egypt	2023	A	

703	PP663055	FVMCU (27)	Felis catus	Egypt	2023	A
704	PP663056	FVMCU (28)	Felis catus	Egypt	2023	A
705	PP663057	FVMCU (29)	Felis catus	Egypt	2023	A
706	PP663058	FVMCU (30)	Felis catus	Egypt	2023	A
707	PP663059	FVMCU (31)	Felis catus	Egypt	2023	A
708	PP663060	FVMCU (32)	Felis catus	Egypt	2023	A
709	PP663061	FVMCU (44)	Felis catus	Egypt	2023	A
710	PP663062	FVMCU (51)	Felis catus	Egypt	2023	A
711	PP663063	FVMCU (53)	Felis catus	Egypt	2023	A
712	PP663064	FVMCU (59)	Felis catus	Egypt	2023	A
713	PP663065	FVMCU (70)	Felis catus	Egypt	2023	A
714	PP663066	FVMCU (71)	Felis catus	Egypt	2023	A
715	PP663067	FVMCU (81)	Felis catus	Egypt	2023	A
716	PP663068	FVMCU (82)	Felis catus	Egypt	2023	A
717	PP663069	FVMCU (90)	Felis catus	Egypt	2023	A
718	PP663070	FVMCU (100)	Felis catus	Egypt	2023	A
719	PP663071	FVMCU (102)	Felis catus	Egypt	2023	A
720	PP663072	FVMCU (103)	Felis catus	Egypt	2023	A
721	PP663073	FVMCU (104)	Felis catus	Egypt	2023	A
722	PP781517	CTU-CT01	Felis catus	Viet Nam	2023	S
723	PP781518	CTU-CT02	Felis catus	Viet Nam	2023	S
724	PP781519	CTU-CT03	Felis catus	Viet Nam	2023	S
725	PP781520	CTU-CT04	Felis catus	Viet Nam	2023	A
726	PP781521	CTU-CT05	Felis catus	Viet Nam	2023	S
727	PP781522	CTU-CT06	Felis catus	Viet Nam	2023	S
728	PP781523	CTU-CT07	Felis catus	Viet Nam	2023	S
729	PP781524	CTU-CT08	Felis catus	Viet Nam	2023	S
730	PP781525	CTU-TV01	Felis catus	Viet Nam	2023	S
731	PP781526	CTU-TV02	Felis catus	Viet Nam	2023	A
732	PP781527	CTU-TV03	Felis catus	Viet Nam	2023	S
733	PP781528	CTU-TV04	Felis catus	Viet Nam	2023	S
734	PP781529	CTU-TV05	Felis catus	Viet Nam	2023	S

735	PP781530	CTU-TV06	Felis catus	Viet Nam	2023	S
736	PP781531	CTU-TV07	Felis catus	Viet Nam	2023	S
737	PP781532	CTU-TV08	Felis catus	Viet Nam	2023	A
738	OR652079	CTU/FVM-TV005	Felis catus	Viet Nam	2023	A
739	OR652080	CTU/FVM-TV004	Felis catus	Viet Nam	2023	A
740	OR652081	CTU/FVM-TV003	Felis catus	Viet Nam	2023	A
741	OR652082	CTU/FVM-TV002	Felis catus	Viet Nam	2023	A
742	OR652083	CTU/FVM-TV001	Felis catus	Viet Nam	2023	A
743	OR399569	FPV-109	Felis catus	China/Jiangsu	2023	A
744	OR399570	FPV-111	Felis catus	China/Jiangsu	2023	A
745	OR399571	FPV-132	Felis catus	China/Jiangsu	2023	A
746	OR399572	FPV-133	Felis catus	China/Jiangsu	2023	S
747	OR399573	FPV-141	Felis catus	China/Jiangsu	2023	S
748	OR399574	FPV-143	Felis catus	China/Jiangsu	2023	S
749	OR399575	FPV-149	Felis catus	China/Jiangsu	2023	S
750	PP619442	F6-2/NJ2304	Felis catus	China/Jiangsu	2023	S
751	PP619441	F24-1/YZ2309	Felis catus	China/Jiangsu	2023	S
752	PP619440	F22-1/YZ2309	Felis catus	China/Jiangsu	2023	S
753	PP619439	F16-1/NJ2307	Felis catus	China/Jiangsu	2023	S
754	PP619437	F11-1/NJ2304	Felis catus	China/Jiangsu	2023	S
755	PP619436	F10-1/NJ2304	Felis catus	China/Jiangsu	2023	S
756	PP619435	F2-1/NJ2304	Felis catus	China/Jiangsu	2023	S
757	PP619438	F14-1/HZ2304	Felis catus	China/Zhejiang	2023	S
758	PP336908	CVASU/BD/50	Felis catus	Bangladesh	2023	A
759	PP738175	Cat-6	Felis catus	Chin/Xinjiang	2023	S
760	PQ212863	XJ-SHZ-1	Felis catus	Chin/Xinjiang	2023	S
761	PQ212864	XJ-SHZ-3	Felis catus	Chin/Xinjiang	2023	S
762	PQ212865	XJ-SHZ-4	Felis catus	Chin/Xinjiang	2023	S
763	PQ212866	XJ-URC-5	Felis catus	Chin/Xinjiang	2023	S
764	PQ212867	XJ-URC-6	Felis catus	Chin/Xinjiang	2023	S
765	PQ212868	XJ-URC-7	Felis catus	Chin/Xinjiang	2023	S
766	PQ212869	XJ-URC-8	Felis catus	Chin/Xinjiang	2023	A

767	PQ227071	XJ-SHZ-2	Felis catus	Chin/Xinjiang	2023	S	
768	OR602718	ITA/2023/hystrix/213	Hystrix cristata	Italy	2023	A	
769	OR602717	ITA/2023/bear/74	Ursus arctos marsicanus	Italy	2023	A	
770	PP663074	FVMCU (107)	Felis catus	Egypt	2024	A	

Figure S1 WB analysis of FPLV-VLP（$NaHCO_3$-treated，Ultra-filtration）

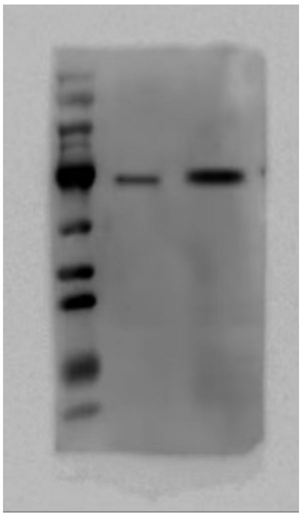

Supplement: Supplementary file 1 [file vetsci-12-00503-s001.zip › vetsci-3574053-supplementary.pdf]
